# Conservation of the Rare and Endangered Vascular Plants in the Mining and Tourism Area: Khibiny Mountains, Murmansk Region, Russia

**DOI:** 10.3390/plants13091180

**Published:** 2024-04-23

**Authors:** Eugene A. Borovichev, Mikhail N. Kozhin, Natalia E. Koroleva, Olga V. Petrova, Diana R. Akhmerova, Maria V. Shulina

**Affiliations:** 1 Avrorin Polar-Alpine Botanical Garden-Institute, Kola Science Center, Russian Academy of Sciences, Apatity 184209, Russia; m.kozhin@ksc.ru (M.N.K.); flora012011@yandex.ru (N.E.K.); olechka.v.petrova@gmail.com (O.V.P.); 2 Institute of Industrial Ecology Problems in the North, Kola Science Center, Russian Academy of Sciences, Apatity 184209, Russia; diana.008@mail.ru (D.R.A.); mshulina@ya.ru (M.V.S.)

**Keywords:** Arctic zone of Russia, biodiversity, environmental protection, mining complex, protected areas

## Abstract

The Khibiny Mountains (hereafter called Khibiny Mts.) are one of the most urbanized and industrialized regions in the Russian Arctic. There are combined a developed mining complex, elaborate infrastructure, a well-known tourist resort, and a large population, all amidst an exceptionally rich biodiversity of plants. In this study, we analyzed the current knowledge of the spatial distribution of rare and endangered vascular plants and vegetation and the impacts of human activities on these ecosystems. Approximately 28% of the protected vascular plant species in the Murmansk Region were registered within the confines of the Khibiny Mts. In particular, although only a handful of protected species had a widespread presence, most rare species were confined to the southern reaches of the mountain range, with only a select few extending into other parts. *Papaver lapponicum* was the only species that thrived across the entire territory, including industrial areas. The studied territory contained nine specially protected areas spanning 123,220 hectares. Nature monuments adjacent to mining sites and urban centers play an important role in preserving regional biodiversity. However, the expansion of the mining industry, alongside deforestation and wildfires, poses considerable threats to the biodiversity of the Khibiny Mts. A comprehensive biodiversity conservation strategy implemented in this region balances the local and expansive territorial protection of rare species and habitats, ensuring environmental preservation while facilitating social and economic progress, a noteworthy example of environmental protection in the Arctic.

## 1. Introduction

Biodiversity is the backbone of human well-being and the environmental cornerstone of ecological safety [1]. Mineral resources are found in all significant biodiversity areas [2,3,4,5]. Therefore, mining threatens biodiversity and ecosystems locally and globally [2]. The relentless exploitation of natural resources and environmental changes has rapidly decreased biodiversity, especially in the Arctic [6,7]. The establishment of protected areas (PAs) is a critical mechanism to protect valuable habitats and vulnerable species [8,9]. The increasing demand for natural resources drives a relentless expansion of land use and land cover, prompting the adoption of site-specific conservation strategies and the creation of various PAs [10]. The concept of “protected area frontier” [11] is a response to the alarming loss of natural and semi-natural habitats in the human-impacted environment.

**Figure 1:**
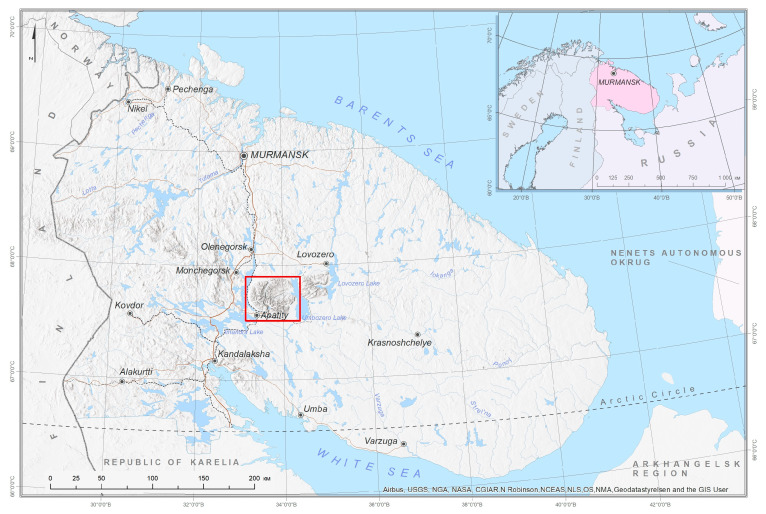
Khibiny Mountains, Murmansk Region, Russia. The explored area is shown in a red rectangle.

The Khibiny Mts., with altitudes from 130 to 1200 m a. s. l., are located in the western part of the Kola Peninsula, NW Russia, and occupy 1300 km^2^ (Figure 1). Geologically, the Khibiny Mts. trace their origins to ancient volcanic intrusion and represent significant geological structures in the Murmansk Region. The region contains the world’s largest apatite–nepheline deposits, exploited by two prominent mining entities, Apatit JSC and North-Western Phosphorus Company. Currently, the industrial and transportation hub is located in the southeastern part of the Khibiny Mts. and adjoining plains, boasting ore deposits, mining and processing enterprises, well-developed infrastructure, agricultural industry, and two towns, Kirovsk and Apatity, with a combined population exceeding 80,000 individuals. Mining operations are crucial for regional and national economic vitality, as well as the welfare of local communities. However, the intensive mining activities have significantly affected the area’s natural environment, resulting in considerable ecological degradation.

Tourism in the Khibiny Mts. exhibits distinct seasonality, with winter offering opportunities for mountain and cross-country skiing, snowmobile tours, and excursions on snowshoes. In contrast, summer is ideal for trekking, hiking, cycling, mountaineering, and mineralogical and scientific–educational tours. Notably, the summer activities are predominantly localized to the Khibiny National Park and Polar-alpine Botanical Garn-Institute. According to data from the Tourism Committee of the Murmansk Region, the influx of tourists to the Khibiny Mts. demonstrates a steady annual increase, with visitor numbers reaching 94,500 in 2021 and increasing to 111,100 in 2022.

The Khibiny Mts. are a natural laboratory perfect for studying Arctic biodiversity, post-glacial biogeography, and evolution. The complex patterns of plant species richness in this region are shaped by many factors, including the harsh and heterogeneous environment, Holocene climatic variations, and diverse histories and vectors of post-glacial plant dispersion. Notably, the southern and southeastern parts of the Khibiny Mts. contain diverse habitats and plant species compared with the adjacent plains, hosting numerous rare and protected species alongside several designated and proposed PAs. While the flora of the Khibiny Mts. has been extensively studied, primarily within the frameworks of several flora and vegetation-based research and conservation projects [12,13,14,15]. There remains a noticeable lack of data concerning mining-related threats to biodiversity, with less than 1% of papers in leading conservation journals addressing this issue [16].

In this study, we analyzed the current distribution of rare and endangered vascular plants and vegetation in the Khibiny Mts., shedding light on the impacts of human activities, in one of the most urbanized and industrialized areas not only in the Murmansk Region but also across the broader expanse of the Russian Arctic.

## 2. Results and Discussion

### 2.1. Distribution of Protected Species

**Figure 2:**
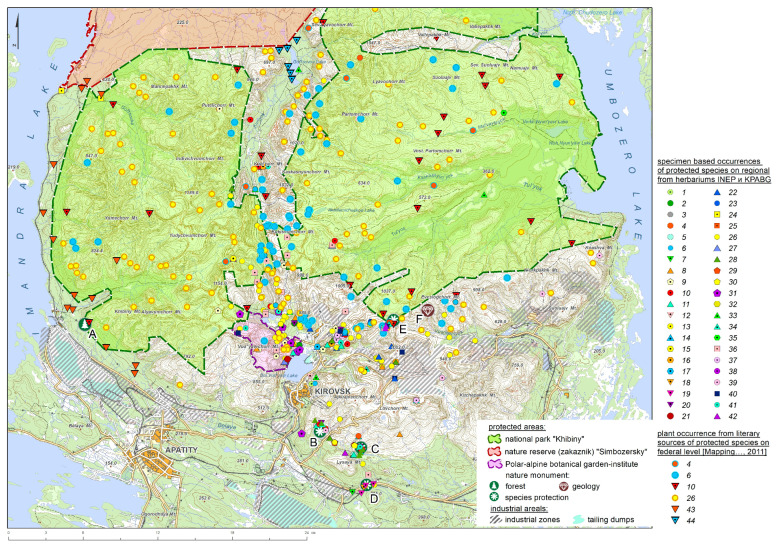
Protected areas and occurrences of protected species in the Khibiny Mountains, Murmansk Region, Russia. Protected species: 1—*Alchemilla alpina* L.; 2—*Alchemilla transpolaris* Juz.; 3—*Anthyllis vulneraria* subsp. *lapponica* (Hyl.) Jalas; 4—*Arnica angustifolia* subsp. *alpina* (L.) I. K. Ferguson; 5—*Asplenium viride* Huds.; 6—*Ranunculus glacialis* L.; 7—*Botrychium lanceolatum* (S. G. Gmel.) Ångstr.; 8—*Carex glacialis* Mack.; 9—*Cassiope tetragona* (L.) D. Don; 10—*Cotoneaster cinnabarinus* Juz.; 11—*Cryptogramma crispa* (L.) R. Br.; 12—*Dactylorhiza maculata* subsp. *fuchsia* (Druce) Hyl.; 13—*Dactylorhiza incarnate* (L.) Soó; 14—*Diplazium sibiricum* (Turcz. ex Kunze) Sa. Kurata; 15—*Draba fladnizensis* Wulfen; 16—*Draba lacteal* Adams; 17—*Draba norvegica* Gunnerus; 18—*Epilobium alsinifolium* Vill.; 19—*Epilobium davuricum* Fisch. ex Hornem.; 20—*Epilobium lactiflorum* Hausskn.; 21—*Epipactis atrorubens* (Hoffm.) Besser; 22—*Erigeron borealis* Simmons; 23—*Gentiana nivalis* L.; 24—*Gypsophila fastigiata* L.; 25—*Hieracium furvescens* (Dahlst.) Omang; 26—*Papaver lapponicum* (Tolm.) Nordh.; 27—*Pilosella arctogena* (Norrl.) Schljakov; 28—*Polystichum lonchitis* (L.) Roth; 29—*Potentilla nivea* L.; 30—*Potentilla chamissonis* Hultén; 31—*Pseudorchis albida* (L.) Á. Löve & D. Löve; 32—*Ranunculus sulphureus* Sol.; 33—*Salix arbuscula* L.; 34—*Salix arctica* Pall.; 35—*Micranthes hieraciifolia* (Waldst. & Kit. ex Willd.) Haw.; 36—*Micranthes tenuis* (Wahlenb.) Small; 37—*Taraxacum nivale* Lange ex Kihlm.; 38—*Taraxacum simulum* Brenner; 39—*Thymus serpyllum* subsp. *Tanaensis* (Hyl.) Jalas; 40—*Koeleria spicata* (L.) Barberá et al.; 41—*Veronica fruticans* Jacq.; 42—*Woodsia glabella* R. Br.; 43—*Calypso bulbosa* (L.) Oakes; 44—*Isoetes lacustris* L. Forest nature monument: A—Siberian pines and larches near the Khibiny Station. Botanical nature monuments: B—Aikuaivenchorr Gorge; C—Cryptogram Gorge; D—Eutrophic FenForest nature monument; E—Juksporrlak. Geological nature monument: F—Astrophyllites of Eveslogchorr Mt.

According to our data, 429 native vascular plants have been registered in the Khibiny Mts., of which 32 species are listed in the regional Red Data Book [15], composing 28% of all protected species in the Murmansk Region. However, the distribution of these species across the Khibiny Mts. is not uniform (Figure 2; Appendix A). Among them, only *Papaver lapponicum* thrives across the entire territory of the Khibiny Mts., inhabiting rocky places ranging from the belt of goltzy deserts to the forest belt. Despite its widespread presence, this species holds a high conservation status and is included in regional and federal Red Data Books [17].

Four additional species commonly occur in the Khibiny Mts. (Figure 2). *Epilobium lactiflorum* and *Polystichum lonchitis* are typically encountered in small populations across various areas of the Khibiny Mts., primarily within the belts of birch krummholz and mountain tundra. These species hold regional conservation status and are confined to grassland communities (including those located on rocks) and birch krummholz throughout the Khibiny Mts. *Ranunculus glacialis* s.str. exhibits a widespread distribution in small populations across the entire territory of the Khibiny Mts., primarily in the belts of mountain tundra and goltzy deserts. Despite its prevalence within the Khibiny Mts., *Ranunculus glacialis* s.str., owing to its limited occurrence within Russia, has a high conservation status and is included in regional and federal Red Data Books.

Eight species are sporadically encountered in the Khibiny Mts., each demonstrating distinct clear ecological preferences. *Cotoneaster cinnabarinus*, protected at regional and federal levels, typically grows in small groups on south-facing rocks above the timberline in various parts of the Khibiny Mts. It is the sole representative of dogwoods in the Khibiny Mts., albeit two other sporadically occurring *Cotoneaster* species are found in the Murmansk Region. In the Khibiny Mts., *Cotoneaster cinnabarinus* approaches the western limit of its distribution. The remaining species possess solely regional conservation status. *Salix arbuscula* inhabits fine-gravel screes and stream banks in the tundra belt across different areas of the Khibiny Mts., typically in small numbers. *Micranthes tenuis*, *Veronica fruticans*, and *Woodsia glabella* are sparsely distributed, primarily above the timberline, and confined to cracks in calcium-containing rocks. *Cassiope tetragona* typically grows in dwarf shrub–forb–moss tundra, fine-grained soil, and rocks among stones above the timberline. *Pseudorchis albida* is prevalent in boggy moss tundra above the timberline, with the largest number of locations observed in the southern part of the Khibiny Mts. *Cryptogramma crispa* is distributed along rocky outcrops near the tree line, primarily in several locations in the southern Khibiny Mts.

Most protected species are rare in the Khibiny Mts. (42 species). These species are primarily located in the southern Khibiny, attributed to extensive historical exploration, good representation in collections, and current field observations. The southern Khibiny offers favorable conditions for rare plant species characterized by well-exposed mountain slopes and outcrops of various rocks, including calcareous ones. Species such as *Arnica angustifolia* subsp. *alpina*, *Alchemilla alpina*, *Carex glacialis*, *Erigeron borealis*, *Gentiana nivalis*, *Draba alpina*, *D. fladnizensis*, *D. lactea*, *D. norvegica*, *Koeleria spicata*, *Micranthes hieraciifolia*, *Taraxacum nivale*, *T. simulum*, *Potentilla nivea*, and *Ranunculus sulphureus*, among others, are predominantly distributed above the timberline. Additionally, *Alchemilla transpolaris*, *Epipactis atrorubens*, *Hieracium furvescens*, and *Pilosella arctogena* are found in the southern part of the Khibiny Mts., specifically within the belt of birch krummholz. In the foothill region of the southern Khibiny Mts., several protected species, including *Epilobium alsinifolium*, *E. davuricum*, *Dactylorhiza maculata* subsp. *fuchsii*, and *D. incarnata*, are localized near eutrophic mire complexes. Furthermore, *Botrychium lanceolatum* is found only in the southern part of the Khibiny Mts. in a single location in a secondary grassland community.

Three species exhibit distribution patterns distinct from those observed in the southern Khibiny Mts. *Isoetes lacustris*, possessing federal and regional conservation status, is exclusively found in the lakes of the northern part of the Khibiny Mts. *Calypso bulbosa* is confined to the spruce forests of the western part of the Khibiny. *Gypsophila fastigiata*, holding solely regional conservation status, grows on river alluvium in the northwestern part of the Khibiny Mts.

For several species, assessing their distribution patterns proved challenging owing to insufficient information regarding their locations in the Khibiny Mts. *Carex holostoma*, *C. tenuiflora*, *Diplazium sibiricum*, and *Eriophorum gracile* are documented solely through old historical records dating back to the 19th century [18]. *Cystopteris fragilis* subsp. *dickieana* and *Deschampsia glauca* lack distinct taxonomic features [19,20], prompting their recommendation for exclusion from the third edition of the regional Red Data Book. Additionally, *Draba alpina* is reported only in the literature without accompanying voucher specimens or precise location information [21].

One approach to preserving rare and endangered species involves cultivating them outside their natural habitats, a practice known as ex situ conservation, often carried out in botanical gardens. The Polar-Alpine Botanical Garden-Institute serves multiple crucial roles: as a research institute, a repository for globally significant plant collections, and a federally protected natural area. Within the protected grounds of the Botanical Garden, 19 regional red-listed species and three federal red-listed plants have been documented (Table A1). Moreover, the botanical garden’s nurseries cultivate five species listed in the Red Book of Russia and recorded in the Khibiny Mts., *Arnica angustifolia* subsp. *alpina*, *Pseudorchis albida*, *Papaver lapponicum*, *Ranunculus glacialis*, and *Cotoneaster cinnabarinus* (from 1938 to present), as well as *Alchemilla alpina* since 1955.

Thus, in the Khibiny Mts., only a few protected species exhibit broad distribution. In contrast, most rare species are confined to the southern region, with only select species found elsewhere in the mountain range. Notably, the species with the highest occurrence frequency also tend to have the widest distribution.

### 2.2. Endemicity

The vascular plant flora of Fennoscandia is characterized by a low diversity of endemics, primarily consisting of species that migrated to the region since the last glaciation [22]. No local endemics of vascular plants are identified in the Khibiny Mts. However, the region hosts two species with extremely limited distribution. *Taraxacum nivale* is endemic to two mountainous regions of the Kola Peninsula. The dandelion is recorded in multiple contemporary occurrences in the Khibiny Mts. In contrast, a sole historical record from the 19th century indicates its presence in the Lovozero Mts. *Papaver lapponicum*, an endemic in Russian Lapland, is predominantly found in the Khibiny Mts., with occasional occurrences in external locations such as the Lovozero Mts., Monchetundra Mt., and the basin of the Voron’ya River [15].

In the 20th century, the endemic diversity of the Khibiny Mts. was mistakenly overestimated. In 1949, a specimen of *Anthyllis* was collected on Yuksporr Mt., which was later described as a new endemic species, *Anthyllus kuzenevae*, from the Khibiny Mts. [23]. Later, mining activities disrupted a portion of this mountain’s territory. The species remained elusive for many decades, leading to its inclusion in the Red Data Books of the Russian Federation [24] and the Murmansk Region [15] with the status of “extinct”. However, according to Jalas [25], these plants belonged to the borealis group, *Anthyllis vulneraria* subsp. *lapponica*, which is widespread in northern Finland and the southern part of the Murmansk Region. Consequently, “*Anthyllus kuzenevae*” was removed from the list of Red Data Books of the Russian Federation [17] and recommended for exclusion from the Red Data Books of the Murmansk Region.

### 2.3. Habitat Types

In recent decades, habitat typology has undergone significant development worldwide. The EUNIS Habitat Classification is a widely adopted reference framework for assessing European habitat types, considering species composition, vegetation structure, abiotic environment, and geographic location [26]. This classification system has become a pivotal component of the updated version of Resolution 4 of the Bern Convention on the Conservation of European Wildlife and Natural Habitats, the legal foundation for both the Natura 2000 and the Emerald networks.

The IUCN criteria for evaluating threatened ecosystems were established [26], encompassing factors such as a short-term decline in the distribution or ecological function, the historical decline in the distribution or ecological function, small current distribution coupled with a decline in the distribution or ecological function, or existence in very few locations. Additionally, ecosystems with very small current distribution and facing serious potential threats, although lacking evidence of past or current decline in area or function, are also considered. Developing the IUCN Red List of Ecosystems at global, regional, and national levels complements the IUCN Red List of Threatened Species. Neighboring countries of the Murmansk Region, such as Norway and Finland, have issued their own Red Data Books focusing on habitats and ecosystems [26,27]. These publications assess habitat types and assign categories based on the IUCN criteria.

A preliminary original habitat typology for the Khibiny Mts. was established based on vegetation classification using the Braun–Blanquet approach [28,29]. This typology encompasses 52 habitats. In the mountain tundra belt, vegetation diversity primarily depends on topographic features, notably the topographic position, associated snow cover depth, and moisture availability [30,31]. Similarly, moisture levels and substrate characteristics influence the diversity of spruce, pine, and subalpine birch forests [32]. While some identified typological units align with those recognized in the EUNIS Habitat Classification (Table 1), the EUNIS habitat types do not fully capture the breadth of habitat diversity observed in the Khibiny Mts.

Protected vascular plant species were detected in 17 habitat types, classified into six distinct groups (Table 1). Notably, nearly one-third (6 out of 17) of these habitat types comprise rocky areas, reflecting the prevalence of this habitat type in the Khibiny Mts. Additionally, a significant proportion (5 out of 17) of the habitat types represent subalpine and subarctic birch forests, which are associated with the expansive coverage of this zone in the broader Murmansk Region.

In the Khibiny Mts., habitats richest in protected species are typically found on dry rock shelves and slopes and in mesic moss–dwarf shrub tundra. Slightly fewer protected species are found in habitats such as mesic mountain low-herb meadows, springs, sloping fens, mesic stony riverbank alluvia, and pebble alluvia. Notably, *Papaver lapponicum* exhibits the highest activity level (5 points) across three habitat types: screes, mesic stony riverbank alluvia, and pebble alluvia. Five other species display an activity level of 4: *Cotoneaster cinnabarinus* and *Polystichum lonchitis*, typically found in low-herb mountain birch forests on rock slopes and shelves, particularly prevalent on the southern and southwestern mountain slopes; *Cassiope tetragona*, widely distributed and active in mesic moss–dwarf shrub tundra, often dominating in this habitat; *Cryptogramma crispa*, exhibiting high activity in plant communities on dry rock shelves and slopes, despite its limited occurrence in the Khibiny Mts.; and *Ranunculus glacialis* and the abovementioned *Cassiope tetragona*, frequently observed on rock faces with seeping water. Other protected species demonstrate low activity levels within plant communities.

A comprehensive list of rare plant community types meeting either the IUCN criteria or the Russian criteria for assessing valuable and endangered ecosystems [31,32,33] has yet to be compiled for the Khibiny Mts. and the broader Murmansk Region. The approach to assessing and conserving habitats associated with rare plant species differs slightly between Russian and European practices. Upon comparing the original habitat classification of the Khibiny Mts. with the EUNIS classification, we found partial or complete correspondence between eight rare habitat types designated for protection under the Bern Convention (Table 1).

In summary, these habitats and protected species occurrences are predominantly concentrated in the southern region of the Khibiny Mts., likely owing to its unique geological characteristics. Positioned at the interface between the Khibiny alkaline intrusion and the Archaean greenstone belt, this area exhibits a complex mineral composition.

### 2.4. Protected Areas Network

In Russia, the official tools for nature conservation primarily entail the establishment of formal PAs and the implementation of regulations aimed at protecting endangered species within their habitats. According to Russian legislation, the discovery of a species listed in the Red Data Book may lead to the creation of PAs (a long and expensive process) or the exclusion of specific locations from economic activities, particularly in economically significant regions. Regrettably, in the Khibiny Mts., this latter approach has not been utilized over the past two decades.

The Federal Law on Specially Protected Natural Areas [34] in Russia distinguishes seven categories—state nature reserve (zapovednik), national park, natural park, sanctuary (zakaznik), natural monument, botanical garden, and dendrological park. The existing nature conservation network in the Khibiny Mts. comprises nine PAs belonging to four categories (Figure 2, Table 2, and Appendix A). These areas collectively cover 1232.2 km^2^, constituting 5.9% of the total protected area in the Murmansk Region (20,999 km^2^).

The Khibiny National Park holds paramount significance in the conservation of vascular plants because over a third of species with regional conservation status and a fifth of those with federal conservation status are found within its boundaries (Table 2). Encompassing the main part of the mountain ranges, Khibiny National Park boasts a diverse array of habitat types. Equally crucial is the PA of the Polar-Alpine Botanical Garden-Institute, dedicated to protecting rare vascular plants. Despite their modest sizes, specialized botanical nature monuments also play a significant role. Particularly noteworthy are Cryptogram Gorge and Yuksporrlak Nature Monuments, which host the largest number of rare species: each contains 16 species with regional conservation status and 4 and 3 species with federal conservation status, respectively. Fewer species are found in Aikuaivenchorr Gorge (Table 2), primarily concentrated in subalpine birch krummholz and mountain tundra habitats. Collectively, the mountain nature monuments contain 20 regional and 4 federal protected species, outnumbering those found in the Polar-Alpine Botanical Garden-Institute, although its territory is more than four times larger. The botanical nature monument Eutrophic Fen is of particular importance for rare plant conservation because it hosts three species of conservation concern, two of which (*Dactylorhiza incarnata* and *Epilobium davuricum*) are represented only in the Khibiny Mts. This monument was specifically established to protect the mire complex and its characteristic vascular plants.

The remaining PAs in the Khibiny Mts. lack a botanical focus and do not serve as primary sites for vascular plant conservation. Located north of the Khibiny Mts., the Simbozersky State Sanctuary (Zakaznik) was established primarily to protect one of the largest wintering and breeding habitats for elk in the Murmansk Region. Only one species of regional and federal conservation concern, the orchid *Calypso bulbosa*, has been documented within its confines. The nature monument Siberian Pines and Larches near the Khibiny station does not host any protected species and predominantly comprises aged forest stands. Additionally, the geological natural monument Astrophyllites of Mount Eveslogchorr has remained largely unexplored from a botanical perspective until recently. 

The current network of PAs encompasses large territories in the Khibiny Mts. However, it inadequately covers the southern region, where many highly threatened species requiring conservation are concentrated (Figure 2). This area harbors species of conservation concern not found in other PAs in the Khibiny Mts., including *Gentiana nivalis*, *Dactylorhiza maculata* subsp. *fuchsii*, *Draba norvegica*, *Potentilla nivea*, *Platanthera bifolia*, and *Ranunculus sulphureus*. Furthermore, several locations of protected vascular plant species in the Khibiny Mts. are not included in the existing protected area network, such as the locations of *Arnica angustifolia* subsp. *alpina* on Rasvumchorr Mt.

To address these issues, we propose granting botanical natural monument status of regional significance to certain territories. Among these areas is the Gorodskaya Shschel’ Gorge, located within the Kirovsk municipal area, which serves as a popular recreational destination for town residents.

In this area, we documented seven protected plant species (*Ranunculus glacialis*, Epilobium alsinifolium, E. lactiflorum, Papaver lapponicum, Pseudorchis albida, *Woodsia glabella*, and the sole population of *Alchemilla alpina* in the Khibiny Mts.). Another place of rare plant species outside the existing PAs in the Skalnoye and Yuzhnoye Gorges is located in the southern part of the Khibiny Mts. Ten protected plant species (*Carex glacialis*, *Cotoneaster cinnabarinus*, *Epilobium lactiflorum*, *Papaver lapponicum*, *Polystichum lonchitis*, *Ranunculus glacialis*, *Saxifraga tenuis*, *Thymus subarcticus*, *Veronica fruticans*, and *Woodsia glabella*) are recorded for these territories. We propose assigning these territories the status of botanical nature monuments of regional significance. The creation of these two nature monuments is outlined in the concept of the functioning and development of a network of specially protected natural areas of the Murmansk Region until 2025 and beyond, extending to 2035 [36].

### 2.5. Human Impact

The primary threat leading to population decline among protected species in the Khibiny Mts. is attributed to the mining complex operations. Approximately 5% of the entire mountain range is occupied by areas of open pits devoid of vegetation, as well as dumps, roads, and other associated disturbances. This proportion is comparable to that of spruce forests in the Khibiny Mts. Geological exploration and mining activities also constitute the predominant sources of anthropogenic disturbances within the territory of Khibiny National Park. Significant amounts of scrap iron, drill pipes, concrete foundation fragments, household waste, and construction debris are prevalent in areas where drilling operations and abandoned settlements once existed. Additionally, at the Juksporrlak pass, a popular tourist destination, erosion and habitat changes are reducing the populations of protected species such as *Arnica angustifolia* subsp. *alpina* and *Veronica fruticans*. Particularly vulnerable to mechanical impact are non-sod gravel-moving substrates, where *Papaver lapponicum* and *Ranunculus glacialis* inhabit.

The forests covering the slopes of the mountains are designated as protective areas and are protected against industrial logging activities. Currently, almost the entire territory of the Khibiny Mts. has been designated as a protective mountain pre-tundra forest. Consequently, all final logging activities are prohibited. However, small-scale logging operations under the guise of thinning persist, exerting some impact across various parts of the national park, particularly in its western cluster. Since 2012, logging activities have been ongoing in the vicinity of the Khibiny Station, leading to substantial degradation of the valley’s recreational resources. In 2012, a large-scale clearing effort for the development of the Partomchorr deposit, encompassing approximately 120 hectares near the Kunyok River’s lower reaches, resulted in a complete transformation of the forest ecosystem in the Khibiny. Here, pine and spruce mountain forests, with an average age of 250–300 years, were decimated. Much of the felled timber was left unremoved or processed into chips, leading to several fires erupting in subsequent years.

Ski resorts are located outside PAs, and skiing activities inflict minimal damage to the biota and landscapes of the Khibiny Mts. However, during the clearing and leveling of trails for the expansion of the ski complex between 2018 and 2021, significant vegetation and soil cover destruction occurred on the slopes of Aikuaivenchorr Mt., resulting in severe erosion.

The introduction of non-native species has a negative impact on the natural ecosystems of most regions of the world. Due to harsh conditions and isolation, mountains are one of the few ecosystems little affected by plant invasions [37]. At the beginning of the 21st century, more than 260 non-native species were listed for the populated territory of the Khibiny Mts. [38], comprising more than half of all the known species in the Murmansk Region. To date, the invasive status of species for the Khibiny Mts. in particular and the Murmansk Region in general has not been discussed. However, we can already identify non-native species with a clear negative impact, e.g., *Heracleum sosnowskyi* Manden., *Lupinus nootkatensis* Donn ex Sims, and *Salix schwerinii* E. L. Wolf. They are actively spreading across disturbed spaces and penetrating into the natural ecosystems of the Khibiny Mts.

In recent decades, there has been significant development of the concept of a natural–technical or ecological–economic system [39,40], which refers to a combination of natural and man-made objects within a given territory. The area under study can be considered a clear example of such a system, particularly during its industrial development phase, characterized by anthropogenic alterations within a limited (albeit sometimes extensive) area while minimally affecting the surrounding natural vegetation cover of the landscape. In the Khibiny Mts., as early as the 1930s, loggers commenced forest clearance in the river valleys within the mountain range and rafted logs downstream along the Goltsovka and Kuna Rivers. Later, during the 1930s and 1940s, forest fires significantly damaged the forests along the railway near the western slopes of the Khibiny Mts. However, the modern human impact exhibits a more pronounced level of destructiveness.

The tourism and recreational cluster “Khibiny”, encompassing the urban districts of Kirovsk Town and Apatity Town, is one of the most dynamically developing areas in the region. Summer tourism activities are mainly localized at the Khibiny National Park. Established in 2018, the park lacks tourism infrastructure to manage visitor influx and alleviate pressure on valuable natural sites and landscapes. A primary objective for regional and municipal authorities is to distribute tourist traffic evenly throughout the year, ensuring the balanced utilization of accommodation and catering facilities. This approach creates favorable conditions for investors and fosters the continuous development of tourism infrastructure. However, this scenario raises significant concerns, emphasizing the urgent need for effective methodologies to evaluate human impacts and monitor ecosystem health.

Uncontrolled tourism and recreation, particularly activities such as littering, trampling, and fires, pose significant threats to biodiversity and habitat integrity in PAs. While these behaviors may result in erosion and localized habitat transformations, they typically do not lead to the extinction of rare species. However, one notable consequence of unchecked tourism is the depletion of dead wood in old-growth mountain forests because it is used for camps and fires. This depletion is particularly evident in spruce forests in the upper reaches of the Tuliyok and the Kaskasnyunyok Rivers, along the entire valley of the Malaya Belaya River, and along the shores of Maly Vudjavr Lake. Nevertheless, when the conservation protocols of the National Park are upheld, tourism and recreation generally do not lead to the extinction of rare species or a decline in biodiversity. Furthermore, promoting tourism fosters widespread public support for PAs, countering the notion of the mountain range solely serving as a mineral resource base.

## 3. Materials and Methods

The research on the distribution of rare species and valuable habitats in the Khibiny Mts. and adjacent plains spanned from 2010 to 2022. A particular focus was placed on PAs and locations harboring species listed in the Red Data Book of the Murmansk Region [15] at the regional level and in the Red Data Book of the Russian Federation [17] at the federal level. The main findings have been partially published [12,13,14]. Latin plant names are referenced using information from the resource for Euro-Mediterranean plant diversity [41] and Plants of the World Online [42].

To describe the geographical distribution pattern of rare species, we systematically reviewed all available herbarium collections at the Polar-Alpine Botanical Garden-Institute (KPABG) and the Institute of Northern Ecology Problems (INEP) in this region. Through this process, previously unpublished records were identified or confirmed by the authors and subsequently incorporated into the dataset. We employed standard georeferencing protocols owing to the lack of geographic coordinates on many specimen labels collected before the 2010s [43]. This involved referencing topographic maps of the Khibiny Mts. at a scale of 1:100,000, archival materials such as field diaries and reports, and high-resolution satellite imagery. Each species of conservation concern was categorized based on its frequency of occurrence using a three-point scale. Frequently occurring species were documented in over 30 localities, occasional species were found in 10–30 localities, and rare species were recorded in fewer than 10 localities. Estimating locality numbers relied on data from herbarium specimens, literature sources, and information from iNaturalist (https://www.inaturalist.org/, accessed on 10 April 2024).

Habitat types were delineated by considering vegetation and landscape characteristics and named according to prevalent plant communities and landscape positions [31,32,44]. The resultant habitat classification was cross-referenced with the EUNIS 2021 and EUNIS [2006–2019] habitat type classifications and Revised Annex I to Resolution 4 [33].

To evaluate the contribution of rare species across different habitat types, we employed a 6-point activity scale [45], which reflected their constancy and average coverage in plant communities. Rare species seldom attained high scores owing to their limited occurrence. Species with the highest activity (4–5) were those present in half or more of the relevés within a habitat type, forming either extensive (activity 5) or smaller (4) populations. Those with average activity (score 3) were found in half or fewer relevés, with varying degrees of abundance, while species with lower activity (score 2) were less abundant. Species with minimal activity (score 1) were recorded in only a few relevés with small coverage or even less (score 0) when observed once or twice.

To evaluate human impacts, we generated a vector spatial layer depicting residential areas and industrial territories, including waste dumps, using open-access satellite imagery and modern topographic maps of the Murmansk Region. The local human influence was recorded during field surveys conducted in 2010–2022 as a remark to relevés and floristic observations (occurrences).

Thematic maps were created using a vector topographic base at a 1:200,000 scale from the Main Research and Information Computing Center of the Ministry of Natural Resources of the Russian Federation. The boundaries and names of PAs were updated in accordance with modern proposals [13,14]. All cartographic operations were conducted using ArcGIS 10.7.1 software, which is the intellectual property of Esri and was utilized herein under license [46].

## 4. Conclusions

The Khibiny Mts. are some of the most urbanized and industrialized regions within the Murmansk Region and the broader Russian Arctic. Here, a well-developed mining complex and a high population density coexist with natural vegetation boasting remarkably high biodiversity, including various protected plant species. We analyzed the current distribution of rare and endangered vascular plants in the Khibiny Mts.

The digitization of historical herbarium data, particularly concerning protected plant species within extensive mining areas, is one of botany’s most important modern tasks. Upon inventorying collections of protected vascular plant species from the Khibiny Mts. housed in the herbaria of KPABG and INEP, we observed a relatively low representation of modern data. Notably, most collections originate from the southern, most developed and anthropogenically transformed part of the Khibiny Mts.

Establishing PAs is an effective approach to mitigate biodiversity loss in industrialized regions. Notably, large PAs such as the Khibiny National Park, Simbozersky State Sanctuary (Zakaznik), and the Polar-Alpine Botanical Garden-Institute protected area play a crucial role in biodiversity conservation across the main territory of the Khibiny Mts. and in the disturbed areas adjacent to urban centers.

The current network of PAs inadequately covers the southern part of the Khibiny Mts., where many highly threatened species requiring conservation are concentrated. We propose creating botanical natural monument status of regional significance for two territories—«Gorodskaya Shschel’ Gorge» and «Skalnoye and Yuzhnoye Gorges». 

Consequently, the studied territory of the Khibiny Mts. is a notable example of biodiversity conservation at site-specific and landscape levels, which does not interfere with the socio-economic development initiatives. In industrialized areas, particularly in the Arctic, a well-functioning PA system preserves biodiversity, ensures socio-economic stability, and enhances the region’s attractiveness for tourism development.

## Figures and Tables

**Table 1:** Valuable habitat types and distribution of rare species in the Khibiny Mountains.

Valuable Habitat Type	EUNIS Habitat Type Classification [33]	Code and Category of Bern Convention Red List [33]	Rare Species [15,17]	Activity
Coniferous forests				
Old-growth dwarf shrubs, moss spruce, and pine forest	T3F—Dark taiga (partly)	—	*Calypso bulbosa*	1
Subalpine and subarctic birch forests				
Cornus-Myrtillus mountain birch forests	T1C1423—Oro-Scandian bilberry-dwarf cornel birch forests	—	*Alchemilla transpolaris*	1
			*Epipactis atrorubens*	0
Low herb mountain birch forests on rock slopes and shelves	T1C1422—Oro-Scandian bilberry-hairgrass birch forests	—	*Cotoneaster cinnabarinus*	3
			*Epipactis atrorubens*	0
			*Polystichum lonchitis*	3
Moist herb-rich subarctic birch forests	—	—	*Epilobium alsinifolium*	1
Mountain tundra				
Mesic moss–dwarf shrub tundra	S224—Boreo-alpine and arctic heaths	—	*Alchemilla alpina*	1
			*Arnica angustifolia* subsp. *alpina*	1
			*Cassiope tetragona*	3
			*Salix arctica*	0
			*Micranthes hieraciifolia*	0
			*Micranthes tenuis*	1
Mountain meadows and grasslands				
Mesic mountain low-herb meadows	R42—Boreal and arctic acidophilous alpine grassland (partly)	RLE4.3a Least concern (partly)	*Arnica angustifolia* subsp. *alpina*	1
			*Gentiana nivalis*	1
			*Micranthes hieraciifolia*	0
			*Micranthes tenuis*	1
			*Taraxacum simulum*	1
Dry mountain low-herb meadows	R42—Boreal and arctic acidophilous alpine grassland (partly)	RLE4.3a Least concern (partly)	*Veronica fruticans*	1
			*Erigeron borealis*	0
Snow bed low-herb meadows	R41—Snow bed vegetation	–	*Pseudorchis albida*	1–2
			*Ranunculus sulphureus*	0
			*Taraxacum simulum*	1
Mountain mires, bogs and fens				
Spring and sloping fens	D2.2—Poor fens and soft-water spring mires D4.2—Basic mountain flushes and stream sides, with a rich arctic-montane flora	–; RLD4.2 Vulnerable	*Epilobium alsinifolium*	1
			*Epilobium lactiflorum*	2
			*Micranthes hieraciifolia*	0
			*Micranthes tenuis*	1
Mesic and paludified spring banks	C3.55—Sparsely vegetated river gravel banks	Resolution 4	*Epilobium alsinifolium*	1
			*Micranthes hieraciifolia*	0
			*Micranthes tenuis*	1
			*Taraxacum simulum*	1
Mountain rocks				
Dry rock shelves and slopes	U25—Boreal and arctic base-rich scree and block field	RLH2.2 data deficient	*Carex glacialis*	1
			*Cryptogramma crispa*	3
			*Draba fladnizensis*	0
			*Draba lactea*	0
			*Erigeron borealis*	0
			*Thymus serpyllum* subsp. *tanaensis*	0
			*Koeleria spicata*	1
			*Woodsia glabella*	0
Rock faces with seepage water	U35—Boreal and arctic base-rich inland cliff	RLH3.2a data deficient	*Ranunculus glacialis*	3
			*Cassiope tetragona*	3
			*Koeleria spicata*	1
Dry and mesic moss–dwarf shrub cushions on weathered rocks	U25—Boreal and arctic base-rich scree and block field	RLH2.2 data deficient	*Cassiope tetragona*	3
			*Ranunculus sulphureus*	0
			*Thymus serpyllum* subsp. *tanaensis*	0
			*Koeleria spicata*	1
Screes	U21—Boreal and arctic siliceous scree and block field	RLH2.1 Least concern	*Papaver lapponicum*	5
Mesic stony river bank alluvia, pebble alluvia	C3.55	Resolution 4	*Gypsophila fastigiata*	0
			*Papaver lapponicum*	5
			*Salix arbuscula*	1
			*Micranthes tenuis*	1
			*Thymus serpyllum* subsp. *tanaensis*	0
			*Koeleria spicata*	1
Abandoned quarries and mountain roads	U313—Boreal and arctic disused siliceous quarries (partly)	–	*Papaver lapponicum*	5
			*Koeleria spicata*	1
Plain mires and fens				
Intermediate wooded and shrub covered fen/moderately rich lawn fen	C2.111—Fennoscandian mineral-rich springs and spring fens	Resolution 4	*Dactylorhiza incarnata*	1
			*Epilobium alsinifolium*	1
			*Epilobium davuricum*	1

Note: Activity: 5—very active; 4—highly active; 3—moderately active; 2—mildly active; 1—low activity; 0—inactive.

**Table 2:** Baseline information on the protected areas in the Khibiny Mountains and number of vascular plant species from the regional and the federal Red Data Books.

Names of the Protected Areas	Area, ha	Year of Establishment	Number of Species from the Red Data Book of Murmansk Region [15]	Number of Species from the Red Data Book of the Russian Federation [17]
National Park (Category IUCN protected area—II—national park)				
Khibiny	84,804	2018	38	5
Protected territory of the Botanical Garden (Category IUCN protected area—Ib—Wilderness Area + Category IV—habitat or species management area)				
Polar-Alpine Botanical Garden-Institute of Kola Science Centre of RAS	1670	1931	19	3
State Sanctuary (Zakaznik) (Category IUCN protected area—IV—habitat or species management area)				
Simbozersky	35,693	2003	1	1
Botanical Nature Monuments (Category IUCN protected area—III—natural monument or feature)				
Aikuaivenchorr Gorge	170	1980	10	4
Cryptogram Gorge	133.6	1980	16	4
Juksporrlak	87	1980	16	3
Eutrophic Fen	19	1980	3	0
Forest Nature Monument (Category IUCN protected area—III—natural monument or feature)				
Siberian pines and larches near the Khibiny Station	4.6	1980	0	0
Geological Nature Monument (Category IUCN protected area—III—natural monument or feature)				
Astrophyllites of Eveslogchorr Mt.	4	1985	no data	no data

Note: IUCN protected area categories are given according to the Guidelines for Protected Areas Legislation [35].

## Data Availability

The data supporting the reported results can be accessed in a publicly archived dataset of the “L” Information system, available at https://isling.org/ (accessed on 19 February 2024).

## Appendix A

**Table A1:** Protected vascular plant species documented within and outside the designated protected areas in the Khibiny Mountains, Murmansk Region, Russia. Abbreviations: RDBMR—Red Data Book of the Murmansk Region [10]; RDBRF—Red Data Book of the Russian Federation [12]; Out PAs—territories not included in PAs; National Park—Khibiny National Park; Botanical Garden—Polar-Alpine Botanical Garden-Institute of Kola Science Centre of RAS; Simbozersky—Simbozersky State Sanctuary (Zakaznik); Juksporrlak—Juksporrlak Botanical Nature Monument; Aikuaivenchorr—Aikuaivenchorr Gorge Botanical Nature Monument; Cryptogram—Cryptogram Gorge Botanical Nature Monument; Eutrophic Fen—Eutrophic Fen Botanical Nature Monument; ●—occurrence supported by a herbarium specimen; ○—occurrence based on the literature data; †—species locality destroyed; ?—doubtful literary record.

Species	RDBMR [12]	RDBRF [14]	Out PAs	National Park	Botanical Garden	Simbozersky	Juksporrlak	Aikuaivenchorr	Cryptogram	Eutrophic Fen
*Alchemilla alpina* L.	3	3	●							
*Alchemilla transpolaris* Juz.	3	–	●		●					
*Anthyllis vulneraria* subsp. *lapponica* (Hyl.) Jalas	0	–	†							
*Arnica angustifolia* subsp. *alpina* (L.) I.K.Ferguson	1б	2	●	●			●	○	●	
*Asplenium viride* Huds.	3	–						○		
*Botrychium lanceolatum* (S. G. Gmel.) Ångstr.	1a	–	○							
*Calypso bulbosa* (L.) Oakes	1б	3	○	○		○				
*Carex glacialis* Mack.	3	–	●		●		●		●	
*Carex holostoma* Drejer	3	–	?							
*Carex tenuiflora* Wahlenb.	3	–	?							
*Cassiope tetragona* (L.) D. Don	3	–	●	●	●		●	●	●	
*Cotoneaster cinnabarinus* Juz.	3	3	●	●	●			●	●	
*Cryptogramma crispa* (L.) R. Br.	3	–	●				●	●	●	
*Cystopteris fragilis* subsp. *dickieana* (R. Sim) Hook.f.	3	–	?							
*Dactylorhiza incarnata* (L.) Soó	2	–								●
*Dactylorhiza maculata* subsp. *fuchsii* (Druce) Hyl.	4	–	○							
*Deschampsia glauca* Hartm.	3	–	?							
*Diplazium sibiricum* (Turcz. ex Kunze) Sa. Kurata	3	–	?							
*Draba alpina* L.	3	–	?							
*Draba fladnizensis* Wulfen	3	–		●			●		●	
*Draba lactea* Adams	2	–	○	●						
*Draba norvegica* Gunnerus	2	–	○							
*Epilobium alsinifolium* Vill.	3	–	●							●
*Epilobium davuricum* Fisch. ex Hornem.	3	–	?							●
*Epilobium lactiflorum* Hausskn.	3	–	●		●		●	●	●	
*Epipactis atrorubens* (Hoffm.) Besser	1б	–			●					
*Erigeron borealis* Simmons	2	–	●	●	○		●			
*Eriophorum brachyantherum* Trautv. & C. A. Mey.	3	–			?					
*Eriophorum gracile* Roth	3		?							
*Gentiana nivalis* L.	2	–	●							
*Gypsophila fastigiata* L.	2	–	●	●						
*Hieracium furvescens* (Dahlst.) Omang	4	–	○		●					
*Isoetes lacustris* L.	3	3	○							
*Koeleria spicata* (L.) Barberá et al.	3	–	●	●			●		●	
*Micranthes hieraciifolia* (Waldst. & Kit. ex Willd.) Haw.	2	–		●						
*Micranthes tenuis* (Wahlenb.) Small	2	–	●	●	●		●			
*Papaver lapponicum* (Tolm.) Nordh.	2	3	●	●	●		●	●	●	
*Pilosella arctogena* (Norrl.) Schljakov	4	–			●					
*Pinguicula villosa* L.	3	–	?							
*Platanthera bifolia* (L.) Rich.	2	–	○							
*Polystichum lonchitis* (L.) Roth	3	–	●	●	●		○	●	●	
*Potentilla chamissonis* Hultén	3	–	●						●	
*Potentilla nivea* L.	3	–	○							
*Pseudorchis albida* (L.) Á. Löve & D. Löve	3	3	●	●	●				○	
*Ranunculus glacialis* L.	2	3	●	●			●	●	●	
*Ranunculus sulphureus* Sol.	2	–	●							
*Salix arbuscula* L.	3	–	●	●	●		●			
*Salix arctica* Pall.	3	–	●		○					
*Taraxacum nivale* Lange ex Kihlm.	3	–	●	●						
*Taraxacum simulum* Brenner	3	–	●		○					
*Thymus serpyllum* subsp. *tanaensis* (Hyl.) Jalas	3	–	●				●	●	●	
*Veronica fruticans* Jacq.	3	–	●	●	●		●		●	
*Woodsia glabella* R. Br.	3	–	●	●			●		●	

Note: Category of RDBRF [14]—2 (species with declining numbers), 3 (rare species); RDBMR [12]—0 (species probably extinct in the region), 1a (in critical condition, under immediate threat of extinction), 1b (in a dangerous condition, endangered), 2(vulnerable species), 3 (rare species).
